# The impact of early-years provision in Children’s Centres (EPICC) on child cognitive and socio-emotional development: study protocol for a randomised controlled trial

**DOI:** 10.1186/s13063-018-2700-x

**Published:** 2018-08-22

**Authors:** Lynne Murray, Susie Jennings, Alicia Mortimer, Amber Prout, Edward Melhuish, Claire Hughes, John Duncan, Joni Holmes, Corinne Dishington, Peter J. Cooper

**Affiliations:** 1 University of Reading, Reading, UK; 2 Stellenbosch University, and the University of Cape Town, Stellenbosch, South Africa; 3 University of Oxford, Oxford, UK; 4 University of Cambridge, Cambridge, UK; 5 MRC Cognition and Brain Sciences Unit, Cambridge, UK; 6 Reading Borough Council, Reading, UK

**Keywords:** Early child development, Cognitive development, Social development, Emotion regulation, Executive function, Parenting intervention, Book-sharing, Dialogic reading, Children’s Centres

## Abstract

**Background:**

There are marked disparities between pre-school children in key skills affecting school readiness, disparities that commonly persist and influence children’s later academic achievements, employment, and adjustment. Much of this disparity is linked to socio-economic disadvantage and its impact on the home learning environment. Children’s Centres are an ideal context in which to implement and evaluate programmes to address this problem. They principally serve the 30% worst areas on the Indices of Deprivation Affecting Children, providing for families from the antenatal period up to age 5 years, aiming to promote parenting skills and provide care for children.

**Methods:**

We are conducting a randomised controlled trial, based in Children Centres, to evaluate a parenting intervention for caregivers of children between 28 and 45 months of age. The intervention provides training to parents in dialogic book-sharing. The training is run by a facilitator who sees parents in small groups, on a weekly basis over 7 weeks. The study is a cluster randomised controlled trial. Twelve of the Children’s Centres in the town of Reading in the UK have been randomly assigned to an index or control condition. The primary outcome is child cognition (language, attention, and executive function); and secondary outcomes are child social development, behaviour problems, and emotion regulation, parenting during book-sharing and problem solving and parental child behaviour management strategies. Data are collected at baseline, post-intervention and 4–6 months post-intervention.

**Discussion:**

The Impact of Early-years Provision in Children’s Centres trial (EPICC) aims to evaluate the impact of an early parenting intervention on several key risk factors for compromised child development, including aspects of parenting and child cognition, social development, behaviour problems and emotion regulation. The study is being carried out in Children’s Centres, which largely serve the most disadvantaged families in the UK. Since the intervention is brief and, with modest levels of training, readily deliverable within Children’s Centres and similar early childcare provision centres, demonstration that it is of benefit to child cognition, socio-emotional development and behaviour would be important.

**Trial registration:**

ISRCTN Registry, ISRCTN28513611. Registered on 28 March 2017.

This is version 1 of the protocol for the EPICC trial.

**Electronic supplementary material:**

The online version of this article (10.1186/s13063-018-2700-x) contains supplementary material, which is available to authorized users.

## Background

There are marked disparities between pre-school children in key skills affecting school readiness (e.g., language, attention, regulation of behaviour and emotions and social relationships) [1, 2]. Much of this disparity is linked to socio-economic disadvantage and its impact on the home learning environment [3–5]. These early childhood effects of disadvantage are important, as they commonly persist and influence children’s later academic achievements, employment and adjustment, thereby perpetuating inter-generational cycles of disadvantage [6, 7]. In the UK, Children’s Centres represent an ideal context in which to implement and evaluate programmes that could address this problem. They were initially established to serve the 30% worst areas on the Indices of Deprivation Affecting Children (IDACI), with wider roll out subsequently. They provide for families from the antenatal period up to age 5 years, aiming to promote parenting skills and provide care for children. Research shows that such pre-school provision can be of particular benefit to children’s later performance and functioning at school when staff are highly trained and support parents’ involvement in their children’s learning at home [8]. One parenting skill that stands to be of particular benefit to children’s development is “dialogic reading”, or “dialogic book-sharing”. This is a method of supporting an infant or young child with a picture book in a way that sensitively follows and supports the child’s interests and engages them actively in a reciprocal interaction. Parental reading or sharing books with children is one of the best predictors of children’s educational outcomes, even when account is taken of family factors like social class and parental education (e.g., [9]). Importantly, disadvantaged parents are less likely than others to share books with their children, and when they do, they tend not to use the “dialogic” techniques that are particularly helpful to child development, and therefore they may be especially likely to benefit from supportive guidance in using these techniques. Indeed, the evidence (principally from North America) clearly shows that it is possible to train parents in good dialogic book-sharing practices, using relatively brief interventions, with consequent benefits for their children’s development [10–12]. Consistent with these previous intervention studies, in a deprived South African population we have shown that our dialogic book-sharing training programme brings about significant benefits to parental sensitivity and reciprocity whilst sharing picture books, and to the child’s attention and receptive and expressive language [13, 14]. Importantly, and extending previous research, we found these gains in child development to be mediated by the improvements in parenting [15]. Further, recent examination of our data indicates that the intervention is especially beneficial for children who are the most disadvantaged.

In our South Africa study we also found, in preliminary analyses, significant benefit of training in dialogic book-sharing to young children’s social understanding and empathy [15]. In order to enhance the benefit of the programme to these child outcomes, we have further integrated into our training programme key principles arising from evidence from naturalistic, observational studies concerning the associations between specific dimensions of parental talk (including during book-sharing), and child socio-emotional development [16]. For example, research shows that when parents talk to their children about emotions and mental states, something which occurs more during book-sharing than in other contexts, children have better emotional understanding and skills in theory of mind, with these benefits even evident in their peer relationships [17–19]. Based on such findings, we have developed our dialogic book-sharing programme beyond the standard focus on cognitive and language outcomes to support parents to use the specific techniques that additionally promote child socio-emotional understanding and pro-social behaviour. This involves careful selection of books that afford parent-child talk about emotions and mental states (e.g., intentions, beliefs and perspectives) and relationships, along with accompanying guidance for parents in how best to support their child’s awareness and understanding of such content. Importantly, we also prioritise books that are largely free of text, in order to maximise free-ranging discussion of the picture content, and to facilitate the use of our programme’s books by parents who may not be literate themselves. We have also incorporated elements into the programme to promote executive functioning skills (e.g., making comparisons, highlighting mutually exclusive relationships, linking elements of depicted content together and to the overall story line) [20]. These original contributions to standard book-sharing practice stand to be of considerable potential benefit to children’s capacity to adjust to the multiple socio-emotional demands of the school environment, adding benefits to the linguistic and cognitive skills previously established to be associated with parental training in book-sharing.

Given the accumulation of research showing the benefits of book-sharing, it is striking that no systematic evaluation of training parents in such structured dialogic book-sharing programmes has been conducted in the UK. Indeed, to our knowledge, although the provision of books for young children and encouragement to parents to share them with their children is widespread, no such evidence-based, systematic and structured training programme is currently delivered in UK Children’s Centres or other UK contexts attempting to serve the needs of more deprived families.

**Fig. 1:**
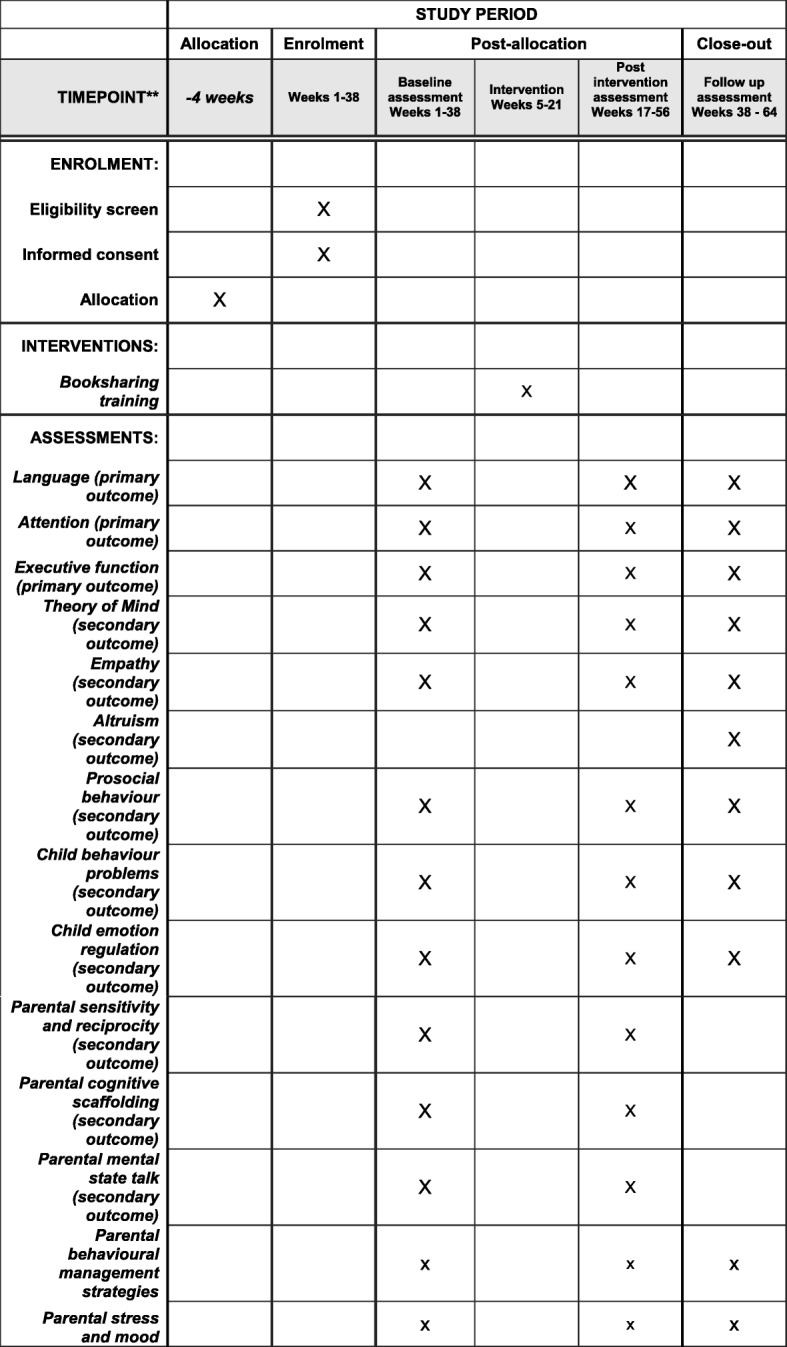
Schedule of enrolment, interventions and assessments*. *Recommended content can be displayed using various schematic formats. See Additional file 1 SPIRIT 2013 Explanation and Elaboration for examples from protocols. **List specific timepoints in this row

We are conducting and evaluating our programme in the UK context. Accordingly, we secured the collaboration of the Children Centres in the town of Reading, carried out pilot work, and established the programme’s acceptability to both staff and parents. We are now delivering the programme to parents in these Children’s Centres, and evaluating the impact on parenting and child cognition, socio-emotional development, and behaviour, both following the intervention and at follow up 4–6 months later. The study time line is shown in Fig. 1.

### Current trial

The study aims are to determine, via a randomised controlled trial (RCT), the impact within Children’s Centres, of providing carers with training in supportive and reciprocal dialogic book-sharing with their young children. In particular, we aim to determine the impact of the training on child cognitive development (primary outcome), on child social development, behaviour problems and emotional regulation (secondary outcomes), and on parenting (secondary outcome).

## Methods

### Study design

The study is a cluster RCT. Reading has 13 Children’s Centres, but one is an outlier in terms of the socio-demographic profile of both ward and attenders. The remaining 12 Centres have been randomly assigned to the index condition (6 Centres), which is receiving training in dialogic book-sharing, and a control condition (6 Centres) receiving the normal input from the Children’s Centres. Data are being collected at baseline, post-intervention, and at 4–6 month follow up.

#### Hypotheses

##### Primary hypothesis

Compared to control group children whose carers receive no additional intervention, children whose carers receive the programme will show evidence of significantly better cognitive outcomes (i.e., observed language, sustained attention and executive function, and parent report).

##### Secondary hypotheses

Compared to control group children, intervention group children will evidence significantly:

1. Better social development (observed theory of mind and emotional understanding, empathy and altruism, and parent report);
2. Fewer behavioural problems (observed defiance and parent report);
3. Better emotional regulation (observed behaviour under challenge and parent report); and compared to control group caregivers, caregivers who receive the programme will evidence significantly -
4. More sensitivity, reciprocity, mental state talk, and cognitive scaffolding with their children (observed behaviours);
5. Better behavioural management strategies (observed behaviour and parent report); and improvement in -
6. Child cognition will be mediated by enhancement of caregiver sensitivity, reciprocity, and cognitive scaffolding;
7. Child social development will be mediated by enhancement of caregiver sensitivity, reciprocity and mental state talk; and
8. Child behavioural problems and emotional regulation will be mediated by enhancement of sensitivity, reciprocity, mental state talk and behaviour management strategies.

### Collaboration

The EPICC study is a collaborative project between the University of Reading, Oxford University, Cambridge University and the Reading Borough Council. The project is funded by the Nuffield Foundation, with further support from the Medical Research Council (MRC) Cognition and Brain Sciences Unit. The Funders have no involvement in the study design or implementation.

### Study setting

The study is being conducted in Children’s Centres in the town of Reading in the UK. The Reading population is representative of the general UK population, including its multi-ethnic communities and significant areas of deprivation. The local authority is strongly committed to Children’s Centres and to the promotion of the development of the under-fives. It is notable that attendance at Children Centres among the most deprived segments of the Reading population has consistently been maintained right through children’s first 3–4 years, rather than dropping off as pupil premiums become available for parents to use in other child care contexts, as occurs elsewhere in the country [21].

### Eligibility criteria

#### Sample

The sample comprises carers (mainly mothers) of children attending the Reading Children Centres, whose children are aged 28–45 months at recruitment and where English is spoken at home. There are 3074 children under 5 years of age living in Reading within the 20% most deprived local super output areas (LSOAs) [22], representing 25% of all Reading children of this age. The 12 participating Children’s Centres in Reading are situated in these areas. They target the most vulnerable families in these most deprived communities, and attendance from this group is consistently over 70% at all Children’s Centres, with these attendees representing the vast majority of Children’s Centre clientele, ensuring socio-demographic homogeneity of the appropriate target population across centres (intra-cluster correlation estimate = 0.04).

The primary outcome in the EPICC trial is child cognition (i.e., language, attention and executive function). Since only one study has examined the impact of book-sharing training on executive function [20], and only one trial has reported an outcome for attention [14], we must calculate sample size from the language variable. The most recent meta-analysis [23] included the 10 RCTs of book-sharing interventions that targeted parents of pre-school children and reported the outcome of child expressive language. Although all but one of these studies showed positive benefit to the children’s language (the exception was one that comprised just three 5-min sessions [24]), there was considerable variability in the nature and duration of the intervention programmes, and there was significant variability in terms of child outcome [23]. The overall effect size for expressive language in the 10 studies was 0.57. However, it is too conservative to base the sample size for the current trial on the effect size for all 10 studies, as the average effect size was 0.88 for the three trials that used a group format of dialogic reading instruction of the form to be used in the current study, and the lowest effect size (0.04) was reported in a study involving just 5-min training sessions [24]. A mid-range medium effect size is therefore justified by the previous data. Using the statistical package R, with *d* = 0.66, within the cluster design, an index and control sample of 96 carers in each are required (with alpha = 0.05 and beta = 0.90). With an addition of 10% to account for sample loss, a total sample of 214 carers is required - i.e., two groups of 107.

### Recruitment

Recruitment began in April 2017 and ended in November 2017. Recruitment took place in two consecutive waves - April to July, and July to November. Recruitment was effected by the study Trial Manager (SJ) approaching parents individually in the Children’s Centres, explaining the study to them, and giving them an information sheet.

### Randomisation

Randomisation of the Children’s Centres to index and control clusters was undertaken by an independent statistician, with minimisation on the index of multiple deprivation (IMD) and ethnic profile of the wards in which each of the Centres is based.

### The intervention, facilitators, training and supervision

#### The training programme

The training programme is for carers and is designed to promote supportive dialogic book-sharing with young children. The programme is based on one trialled in South Africa and found to be highly effective in improving carer book-sharing skills, and to have significant benefit on child development [13–15]. The programme has been piloted in a UK Children’s Centre (Pen Green in Corby) where it was enthusiastically received by both staff and parents. The programme involves parents meeting in small groups (4–6) and receiving instruction from a facilitator over seven, weekly, one-hour sessions. These sessions, which are organised around a “book of the week”, involve a Microsoft PowerPoint presentation with demonstration video clips to illustrate key learning points, with book-sharing skills built incrementally. After the first session, each session begins with a review of participants’ book-sharing experiences during the preceding week. The group session ends with each parent being given the book to take home to share with their child, with encouragement to do so on a daily basis for approximately 10 min. After the one-hour group session, there is a 5–10-min period in which each parent shares the book with their own child, under the support and guidance of the facilitator. The content of the seven sessions of the programme is shown in Table 1. It can be seen that, following an introductory session, there are six substantive sessions, with each session framed around specific learning content (e.g., talking about emotions, or about perspectives). Families keep the books they take home each week, and during the 4–6-month follow up period we send each family a new book on two occasions.

**Table 1:** Intervention session content

Session	Session content
1	**Introduction to book sharing (using** ***Handa’s Surprise*** **by Eileen Browne)** The benefits to child development of book-sharing are explained, and the importance of establishing a book-sharing routine stressed. Basic principles of dialogic reading are outlined, including following the child’s lead, as well as techniques such as pointing and naming and asking “who/what/where” questions to engage the child and encourage dialogue
2	**Elaborating and linking (using** ***Little Helpers*** **by Lynne Murray, Peter Cooper and Lyn Gilbert)** Picking up on the child’s focus of interest and elaborating on it. Making links between the book content and the child’s own experience. Making links between different elements of the book, and their relation to the overall book narrative
3	**Numeracy and comparisons (using** ***Handa’s Hen*** **by Eileen Browne)** Introducing the idea of counting and comparative concepts (e.g., more, less, highest, smallest) and category inclusion and exclusion
4	**Talking about feelings (using** ***Hug*** **by Jez Alborough)** Talking about the feelings of the book characters. Naming feelings and contextualising them. Linking the book characters’ feelings to the child’s own emotional experience
5	**Talking about intentions (using** ***Harry the Dirty Dog*** **by Gene Zion and Margaret Bloy Graham)** Discussing why characters feel the way they do, asking what characters are thinking and intending, encouraging the child to be curious about what will come next in the story
6	**Talking about perspectives (using** ***Harry by the sea*** **by Gene Zion and Margaret Bloy Graham)** Helping the child understand that different people can see things differently, know different things, and feel differently about things
7	**Relationships (using** ***The Wrong Side of the Bed*** **by Edward Ardizzone)** Discussing family relationships, including conflict and resolution

For the current trial, the intervention was delivered by two facilitators - a primary school teacher and an early-years practitioner. They were trained in the intervention during a 3-day workshop run by LM and PJC with the help of David Jeffery, the Chief Executive Officer (CEO) of the Mikhulu Child Development Trust (www.mikhulutrust.org). Both the facilitators ran a practice group before the trial commenced.

Weekly supervision was provided to the facilitators throughout the intervention phase of the study. LM and PJC met with the facilitators for an hour each week to review how the sessions went over the previous week. The two facilitators discussed aspects that went well, challenges, and logistical issues. They also identified any participants who were experiencing difficulties in applying the programme with their child, and discussed the support they may need in their next session. Finally, the group reviewed the attendance records from the past week and discussed plans for catch up sessions with any participant who may have missed sessions. The number of sessions each participant attended was recorded.

### Data collection

#### Data collector training

Two data collectors (AM and AP), both with a Master’s degree in Developmental Psychopathology, were trained in the child assessments and caregiver assessments. Training was held over a one-month period and followed a data collector manual developed by LM and PC. During the three assessment waves, LM and PJC make regular checks through examination of the data to ensure fidelity of assessment administration. Both data collectors are familiar with consent and referral procedures and with how to discuss potentially sensitive topics with caregivers during the assessment.

#### Procedures

All carer/child pairs are assessed on three occasions: at baseline, following the 7-week intervention, and 4–6 months post-intervention. For the baseline assessment, caregivers are contacted by the Trial Manager (SJ) and the study is explained to them. It is emphasised that participation is entirely voluntary and that non-participation will not affect the service they receive from the Children’s Centre. A suitable time for them to come in to the Children’s Centre for assessment by the data collector is arranged. On arrival at the assessment session, consent is explained again and caregivers provide consent for both themselves and their child. Assessments take up to 2.5 h. They comprise specific assessments of the child (e.g., the Early Childhood Vigilance Task), questionnaires completed by the caregiver (e.g., the Strengths and Difficulties Questionnaire), and filming the caregiver and child in interactive tasks (e.g., book-sharing). There are breaks for refreshment, and if the child shows any signs of tiredness or distress, the session is interrupted or, if necessary, terminated. Participants are given a small gratuity for contributing their time to the study. Similar procedures are followed for the subsequent two assessment waves. To prevent assessment bias, assessments of children and caregivers are being carried out blind to group allocation, including explicitly asking participants not to reveal their allocation to the data collectors. All coding of video material is made blind to allocation.

#### Retention

Provisions have been put in place to maximise participant retention. This includes texts and phone calls to remind participants of scheduled assessments.

### Outcomes

For details of outcomes see Table 2

**Table 2:** Study outcomes and measures

				Post allocation	
			Week 1–4	Week 18–22	Week 30–38
Outcomes	Concept	Measures	Baseline	Post	Follow up
Child cognitive development (primary outcome)	Language				
	Expressive	EYT (expressive vocabulary)	X	X	X
		Parent report: CDI	X	X	X
	Receptive	Pre-school CELF-2			X
		Parent report: CDI	X	X	X
	Attention				
		EYT (Go No Go)	X	X	X
		ECVT	X	X	X
		Observation: 3 toys task	X	X	X
		Parent report: SDQ and CSBQ	X	X	X
	Executive function				
	Non-verbal reasoning	WPPSI block design		X	X
	Task shifting	EYT (card sort)			X
	Phonological working memory	Digit span		X	X
		Following instructions		X	X
	Persistence and strategies	Observation: Lab-TAB	X		X
	Inhibition	EYT (Go No Go)	X	X	X
	Self- regulation	Parent report: CSBQ	X	X	X
Child social development (secondary outcome)	Theory of mind and emotional understanding	Observation: ToM package	X	X	X
	Empathy	Observation: help task	X	X	X
	Altruism	Observation: token sharing			X
	Pro-social behaviour	Parent report: SDQ	X	X	X
Child behavioural problems (secondary outcome)		Observation: don't touch task	X	X	X
		Parental report: SDQ	X	X	X
Child emotion regulation (secondary outcome)		Observation: Lab-TAB	X		X
		Parent report: CSBQ	X	X	X
Parental sensitivity and reciprocity (secondary outcome)		Observation: book-sharing	X	X	
Parental cognitive scaffolding (secondary outcome)		Observation: book-sharing and puzzle task	X	X	
Parental mental state talk (secondary outcome)		Book-sharing	X	X	
Parental behavioural management strategies		Observation: don't touch task	X	X	X
		Parent report: Discipline Scale	X	X	X
Parental stress and mood		Parent report: PSI	X	X	X
		Parent report: HADS	X	X	X

*Abbreviations: EYT* Early Years Toolbox, *CDI* Communication Development Inventory, *CEL*F Clinical Evaluation of Language Fundamentals, *ECVT* Early Child Vigilance Task, *SDQ* Strengths and Difficulties Questionnaire, *WPPSI* Wechsler Preschool and Primary Scale of Intelligence, *Lab-TAB* Laboratory Temperament Assessment Battery, *CSBQ* Child Self-Regulation and Behaviour Questionnaire, *PSI* Parenting Stress Index, *HADS* Hospital Anxiety and Depression Scale

#### Assessments

Independent assessments are being made of index and control participants by the two trained data collectors. These assessments are made in the Children’s Centres for the first two assessments (and occasionally in families’ homes) and the final assessment is made at the School of Psychology and Clinical Language Sciences of the University of Reading.

The assessments being made are as follows:

- Child outcomes
  - *Cognitive development (primary outcome)*

*Language*: expressive language is being assessed using the Early Years Toolbox (EYT) [20], administered using iPad technology, and receptive language using the Clinical Evaluation of Language Fundamentals (CELF-2) [25]. A parental report measure of language is also being administered, the Communication Development Inventory (CDI) [26].

*Attention*: this is assessed by deriving an index from the Go-No-Go sub-test of the EYT, by the Early Child Vigilance Task (ECVT) [27], and by observing behaviour in “the three toys” task [13, 28], and by parent report on the Strengths and Difficulties Scale (SDQ) [29] and the Child Self-Regulation and Behaviour Questionnaire (CSBQ) of the EYT [20].

*Executive function*: this is being assessed using the EYT, Block design (WPPSI-IV) [30], Digit span [31], the Following Instructions task [32], and persistence and strategies in the Frustration task of the Laboratory Temperament Assessment Battery (Lab-TAB) [33], and by parent report on the CSBQ [20].

- *Child social development (secondary outcome)*

*Theory of mind and emotional understanding*: this is being assessed using a battery of tasks based on Wellman and Liu [34], Hughes et al. [35] and Denham [36]. These tasks comprise brief, typically puppet-enacted scenarios, during which young children are asked to judge the different perspectives, knowledge, desires and emotions of the characters.

*Empathy*: a “help task” paradigm is being administered in which a researcher feigns experiencing a problem (i.e., being unable to find something that is in view of the child) and the child’s response is observed [15, 37, 38].

*Altruism*: a modified version of an altruism task [39] is being used.

*Prosocial behaviour*: this is being measured by parent report using the SDQ [29].

- *Child behaviour problems (secondary outcome)*

These are being assessed by maternal report and direct observation. For maternal report we are using the SDQ [29]. Child behaviour is also assessed by direct observation during the parent-child interactions in the “don't touch”’ task (in which the child is prohibited from touching attractive toys) [40–42].

- *Emotion regulation (secondary outcome)*

This is being assessed by direct observation using the Distress-Anger/Frustration component of the Lab-TAB for pre-school children, and by parent report on the CSBQ [20].

- Parenting (secondary outcome)

*Sensitivity and reciprocity* [13, 15] and *cognitive scaffolding* are being assessed by direct observation of parent-child interaction in book-sharing and a problem solving task. *Mental state talk* is also assessed during book-sharing.

*Parent behaviour management strategies*: these are being assessed by maternal report and direct observation. Parents complete the Child Rearing Discipline Scale [43], and they are directly observed during the “do not touch” task described above [39, 43].

### Potential moderators

The following variables will be used as potential moderators: family socio-economic status, parental education, monolingualism status, ethnic group, parental mental health (HADS; [44]), stress (PSI [45]), and baseline book-sharing, child sex, age and birth order.

### Data management

Data are submitted to a secure University server. Participants are allocated personal identification numbers that are used in all study records to protect their identity and maintain confidentiality. All child assessments and caregiver-child interaction tasks are video-recorded, for both coding and quality control purposes. Videos are labelled by the assessors and saved on the secure server at the end of each day.

### Data analysis

Data analysis will be completed by a designated statistician independent from study investigators. Group baseline differences will be assessed using the independent samples *t* test, Mann-Whitney U test, and chi-squared test. Analysis of baseline group differences will include socio-demographic data, such as child sex, and household factors (e.g., income, relationship status). The primary and secondary outcomes will be analysed using linear mixed models which can account for clustering of individuals within the Children’s Centre and for repeated assessments within individuals (for outcomes measured at multiple time points). Intervention effects will be assessed post intervention and at follow-up, and will be adjusted for the child’s age, sex and baseline scores (where applicable). Further socio-demographic factors may also be investigated as covariates. If the necessary assumptions of the models do not hold, suitable alternative models will be fitted. Intention-to-treat analysis will be used to examine intervention effects [45]. The amount and pattern of missing data will be examined and will be addressed using multiple imputation where appropriate.

#### Mediator analyses

Mediator analyses will aim to identify active components of the intervention and elucidate the pathways to change. To this end, the following three questions will be examined: whether improvements in maternal sensitivity and reciprocity and cognitive scaffolding mediate improvements in child cognition; whether increases in maternal sensitivity, reciprocity and mental state talk mediate improvements in child social understanding; and whether increases in maternal sensitivity, reciprocity and mental state talk and improved behaviour management strategies mediate improvements in child behaviour, and emotional regulation.

#### Moderator analyses

Moderator analyses will be conducted to investigate whether certain groups respond differently to the intervention. In addition to the potential moderators listed above, we will examine the impact of number of intervention sessions attended. Potential mediators and moderators of the intervention will be examined using mixed linear models or structured equation modelling, as appropriate.

### Trial monitoring

#### Trial Steering Committee

An independent Trial Steering Committee (TSC) monitors the progress of the trial and advises the research team on matters arising during the course of the study. The TSC meets bi-annually. The TSC is chaired by a Professor of Education from Oxford University. Its other members, in addition to the four Principal Investigators (PIs)/co-PIs (LM, PJC, CH, EM) and the Trial Manager (SJ), are a parent and representatives of children’s advocacy groups (Action for Children and the Foundation Years Trust). If the assessors, the facilitators, or the Trial Manager have concerns about the children or the carers, they inform the Reading PIs (LM/PJC) who take appropriate steps to address the problem.

## Discussion

The EPICC trial is an evaluation of an intervention in which carers of young children who are attending a Children’s Centre are provided with training over 7 weeks in dialogic picture-book sharing with their child, following a specific manualised programme. The trial aims to evaluate the impact of this early parenting intervention on child cognition (i.e., language, attention and executive function). Its impact will also be evaluated on child social development (i.e., theory of mind and social understanding, empathy, altruism and pro-social behaviour), behavioural problems, and emotional regulation. Effects on parenting will also be assessed - i.e., sensitivity, reciprocity, cognitive scaffolding, mental state talk, and behavioural management strategies. The study is being carried out in UK Children’s Centres. Since the intervention is brief and readily deliverable with modest levels of training, demonstration of benefit to child cognitive and socio-emotional development would be important. This study is similar in its methodology to a dialogical book-sharing trial currently being conducted in a poor community in South Africa [46].

### Dissemination plans

#### Outcomes, outputs and dissemination

Following receipt of the trial statistical report, we will disseminate the study findings in several ways. We will publish them in major peer reviewed academic journals and in relevant professional journals. We will produce a summary of the project’s objectives, methodologies and key findings, together with recommendations for policy and practice, which will appear on the University of Reading website. We will also write a briefing paper for distribution to the Department for Education (specifically the Minister for Children and his/her policy team), the Local Government Association, Confederation of Scottish Local Authorities, the European Social network and to a range of early years associations/non-governmental organisations (NGOs) (e.g., Save the Children, Barnardo’s, Action for Children, National Childbirth Trust (NCB)) and the media. It is likely that this will lead to press, radio and TV coverage of the findings. We will make oral presentations at psychology, education and professional organisation conferences, both nationally and internationally, and together with our media coverage this will engage the attention of parents and other interested groups. Finally, we will hold an end of study workshop. This workshop will include the PI/co-PIs (LM, PJC, CH, EM) and the Steering Committee members, together with policy makers from the DfE and Local Authorities and academics and practitioners with special interest and expertise in this area. Through the research team’s involvement (and particularly that of EM), with international policy agencies, such as the Organisation for Economic Cooperation and Development (OECD), the WHO, and the European Commission, opportunities for international impact will likely emerge. To ensure the possibility of scale up, LM and PC will provide the dialogic book-sharing training materials to key stake-holder organisations on request and, via the Mikhulu Trust, will support the provision of courses in the training programme and accreditation of trainers.

### Trial status

At the point of submitting this manuscript to the journal (30 October 2017) 212/214 of the sample had been recruited. This paper represents version 1 of the protocol.

## Additional file

Additional file 1: SPIRIT 2013 Checklist: Recommended items to address in a clinical trial protocol and related documents. (DOCX 63 kb)

## Abbreviations

CDI: Communication Development Inventory
CELF: Clinical Evaluation of Language Fundamentals
CEO: Chief Executive Officer
CSBQ: Child Self-Regulation and Behaviour Questionnaire
DfE: Department for Education
ECVT: Early Child Vigilance Task
EPICC: Early-years Provision of Care in Children’s Centres
EYT: Early Years Toolbox
HADS: Hospital Anxiety and Depression Scale
IDACI: Indices of Deprivation Affecting Children
IMD: Index of Multiple Deprivation
ISRCTN: International standard randomised controlled trials number
Lab-TAB: Laboratory Temperament Assessment Battery
LSOA: Local super output area
NCT: National Childbirth Trust
OECD: Organisation for Economic Cooperation and Development
PSI: Parenting Stress Index
RCT: Randomised controlled trial
SDQ: Strengths and Difficulties Questionnaire
TSC: Trial Steering Committee
UREC: University of Reading Research Ethics Committee
WHO: World Health Organisation
WPPSI: Wechsler Preschool and Primary Scale of Intelligence
